# Andrographolide enhances the anti-metastatic effect of radiation in Ras-transformed cells via suppression of ERK–mediated MMP-2 activity

**DOI:** 10.1371/journal.pone.0205666

**Published:** 2018-10-25

**Authors:** Chih-Chia Yu, Chien-An Chen, Shu-Ling Fu, Hon-Yi Lin, Moon-Sing Lee, Wen-Yen Chiou, Yu-Chieh Su, Shih-Kai Hung

**Affiliations:** 1 Department of Radiation Oncology, Dalin Tzu Chi Hospital, Buddhist Tzu Chi Medical Foundation, Chia-Yi, Taiwan, R.O.C; 2 Department of Radiation Oncology, Zhongxing Branch, Taipei City Hospital, Taipei, Taiwan; 3 Institute of Traditional Medicine, National Yang-Ming University, Taipei, Taiwan; 4 School of Medicine, Tzu Chi University, Hualian, Taiwan, R.O.C; 5 Division of Hematology and Oncology, Department of Internal Medicine, Kaohsiung Medical University Hospital, Taiwan; 6 Faculty of Medicine, College of Medicine, Kaohsiung Medical University, Kaohsiung, Taiwan; Duke University School of Medicine, UNITED STATES

## Abstract

**Background:**

Activation of Ras oncogene in human tumors is associated with radiation-associated metastatic potential. Although ionizing radiation is one important method of cancer treatments, it has been shown to enhance matrix metalloproteinases (MMPs) activity and facilitates a more aggressive cancer phenotype. Our previous studies showed that andrographolide with lower dose rates of radiation could inhibit RAS-transformed cancer metastasis *in vivo*; however, the molecular mechanisms are not yet clear. In this study, we aimed to explore the anti-metastatic effect of andrographolide combined with radiation on Ras-transformed cells.

**Methods:**

RAS-transformed cells were treated with andrographolide in the presence or absence of irradiation (2–4 Gy) or angiotensin II to examine cell invasion. *In vivo* tumorigenesis assays were also performed. The MMP-2 activity was detected by using Gelatin zymography. Signal transduction of NF-κB subunit, p65 and phosphor-ERK 1/2, were examined by using Western blotting analysis.

**Results:**

Treatment with andrographolide inhibited migration of Ras-transformed cells. Andrographolide treatment with radiation significantly inhibited cancer metastasis *in vivo*. We found that andrographolide exhibited anti-migration and anti-invasive ability against cancer metastasis via inhibition of MMP2 activity rather than affected MMP-9 and EMT. In addition, combined andrographolide with radiation appeared to be more effective in reducing MMP-2 expression, and this effect was accompanied by suppression of ERK activation that inhibits cancer cell migration and invasion.

**Conclusions:**

These findings suggest that andrographolide enhances the anti-metastatic effect of radiation in Ras-transformed cells via suppression of ERK–mediated MMP-2 activity.

## Introduction

Radiotherapy (RT) is a curative treatment option for some malignant tumors. However, local relapse and distant metastasis are two major causes of RT failure. Tumor metastasis is driven by accumulation of intrinsic and extrinsic alterations in malignant cells that increase motility and invasiveness of the cells, which can allow them to be resistant to cytotoxic therapies.

Matrix metalloproteinases (MMPs) have been considered to increase the metastatic potential of various cancers because they degrade basement membrane, and alter cell-cell and cell-extracellular matrix (ECM) interactions, migration, and angiogenesis [1,2]. MMP-2 (gelatinase A) and -9 (gelatinase B), both of which are cancer-associated, zinc-dependent endopeptidases, have been implicated in playing a significant proteolytic role in cancer invasion and metastasis [3]. Previous studies have indicated that the expression of MMP-2 and MMP-9 are enhanced by radiation in a number of tumor types, and their expressions have been associated with poor prognosis [4–6].

The mitogen-activated kinases (MAPKs) pathway has been previously related with MMPs expression and metastasis progression. In addition, NF-κB has been reported to be involved in regulating MMP-2 and MMP-9 expression [7,8]. Upregulation of MMP-9 has been observed in hepatocellular carcinoma (HCC) exposed to ionizing radiation, and radiation-induced HCC invasiveness is enhanced through the PI3K/AKT/NF-κB signal transduction pathway [6]. It has also been reported that MMP-9 inhibition downregulates radiation-induced NF-κB activity, leading to apoptosis in breast tumors [9]. The activation of MMP-2 has been shown to be a crucial step in tumor invasiveness and is associated with more aggressive phenotypes [10]. MMP-2 siRNA combined with radiation was shown to significantly reduce migration, invasion, growth, and angiogenesis in glioblastoma cells [11].

Andrographolide, a bicyclic diterpenoid lactone, is the primary ingredient of the medicinal herb *Andrographis paniculata*, and it has been reported to have a wide range of biological activities including anti-inflammatory [12], anti-allergic [13], anti-oxidation [14], and anti-cancer effects [15,16]. In addition, it has been demonstrated to have anti-metastatic activity *in vitro* and *in vivo* on various tumors via inhibition of MMP-2 or MMP-9 activity [17–20]. Moreover, andrographolide has been shown to have potent radiosensitizing activity though increased autophagy [21] and apoptosis [22], as well as through an anti-invasion effect [23]. As a result, andrographolide sensitizes tumor cells to the cytotoxic effects of radiation.

Our previous studies have shown that andrographolide produces a radiation-induced cytotoxic effect in Ras-transformed cell *in vitro* and *in vivo* via attenuating NF-κB activity [24]. We also found that andrographolide combined with lower dose-rate radiation synergistically enhances the anti-metastatic effects of Ras-transformed cells in a xenograft mouse model [25]. Although andrographolide may be a promising strategy for enhancing the anti-metastatic effect of radiation, the molecular mechanism in Ras-transformed cells remains to be elucidated. In the present study, we extended our previous study to explore the anti-metastatic effects of andrographolide with RT, and the potential molecular mechanisms involved.

## Materials and methods

### Reagents and chemicals

Andrographolide (Merck Millipore, Darmstadt, Germany) was dissolved in dimethyl sulfoxide (DMSO) as a concentrated stock solution, and stored at −20°C until further use.

### Cell lines and cell culture

Ras-transformed cells were obtained from the laboratory of Shu-Ling Fu. The cell line derived from rat kidney (RK3E/tv-a) was infected with a retrovirus carrying the oncogene Ras as described in a previous study [26]. The cells were regularly maintained in DMEM containing 10% FBS, 100 units/ml penicillin, 100 μg/ml streptomycin, and 3μg/ml puromycin in 10 cm dishes at 37°C in a humidified atmosphere of 5% CO2 and 95% air.

### *In vivo* tumorigenesis assays

The Ras/Luc cell line, which is a Ras-transformed cell line constitutively expressing the luciferase gene, was used in the present study. Briefly, 10^6^ Ras/Luc cells in 100 μl phosphate-buffered saline were injected into the back or tail vein of nude mice. Male 6–8 week-old athymic nude mice (BALB/cAnN.Cg-Foxn1nu/CrlNarl) were obtained from the National Laboratory Animal Center, Taiwan. Mice were maintained under 12h light/dark cycle at room temperature (24±1°C) and 60±5% humidity. Mice were divided into four groups according to the treatment administered: group 1 (n = 12), andrographolide (10 uM); group 2 (n = 12), radiation (2 Gy) and vehicle (100 ul DMSO); group 3 (n = 12), andrographolide (10 uM) plus radiation (2 Gy); and group 4 (n = 12), vehicle (100 ul DMSO). Andrographolide dosage was selected based on a previous *in vivo* study [24]. Oral administration of andrographolide was given 3 hours prior to radiation. An electron linear accelerator (Varian Medical Systems, Palo Alto, CA, USA) was used to apply radiation. At the end of the experiments, all remaining mice were sacrificed by cervical dislocation. All animal protocols were performed according to the instructions issued by the Institutional Animal Care and Use Committee of National Yang-Ming Cheng University (IACUC no.1010611).

### Wound healing assay

A monolayer of cells was grown to confluence in 10 cm plates, and at experimental time zero a scratch was made in each well using a pipette tip. The cells were washed twice with PBS before their subsequent incubation with culture medium in the absence (control) or presence of 10 μM andrographolide. Photos were taken of the scratches at 0 h and 12 h with a digital camera.

### Western blotting

After treatment, cells were harvested and lysed, and protein concentrations were measured by using the Bio-Rad protein assay kit (Bio-Rad, Richmond, CA, USA). All samples were separated on SDS-PAGE gel and transferred to a PVDF membrane (Millipore, Billerica, MA, USA). After blocking the membranes with 5% (w/v) non-fat dry milk in TBS containing 0.1% Tween 20 (TBS-T) for 1 hour at room temperature, they were immunoblotted with the following monoclonal primary antibodies: vimentin, E-cadherin, MMP-2, MMP-9, phosphor-ERK1/2, total-ERK1/2, NF-kB subunit p65, β-actin, and GAPDH (all from Cell Signaling Technology, Beverly, MA,USA). Appropriate horseradish peroxidase-conjugated secondary antibodies, including mouse IgG and rabbit IgG antibodies (Abcam, Cambridge, MA, USA), were applied and incubated for 1 h at room temperature. Specific signals were visualized using a chemiluminescence (ECL) detection kit (Millipore).

### Cell invasion assay

The invasion assay was performed using Millicell Transwell Cell Culture Inserts (8 μm pores, Millipore, Billerica, MA, USA). The cells (2×10^4^ cells/insert) were placed in the upper well of the chamber, and serum-free conditioned medium was placed in the lower well as a chemoattractant. The filter was a polyvinylpyrolidone-free polycarbonate membrane with 8.0 μm pores. The bottom wells of the system were filled with complete medium, and incubated for 48 h at 37°C. The cells from inserts were fixed with 4% paraformaldehyde (PFA) and stained with 0.1% crystal violet. Invasion was quantified by reading the optical density (OD) at 590nm after crystal violet staining.

### Gelatin zymography

Gelatin zymography was carried out by using 10% SDS-polyacrylamide gels containing 0.1% gelatin. After electrophoresis, gels were washed twice with 100 mL distilled water containing 2.5% Triton X-100 for 30 min at room temperature to remove the SDS. The gel was then incubated in 100 mL reaction buffer (50 mM Tris-HCl, pH 7.6, 200 mM NaCl, 5mM CaCl2) overnight at 37°C. Gels were stained with 2.5% Coomassie Brilliant Blue, and enzymatic activity was detected by observing the lack of gelatin protein in sample lanes, represented by a clear band.

### Statistical analysis

All data were presented as the mean ± standard deviation and were compared by using Student’s t-test or ANOVA test. *P* values of < 0.05 were considered as a statistical significance.

## Results

### Andrographolide decreased the Ras-transformed cell migration activity

**Fig 1:**
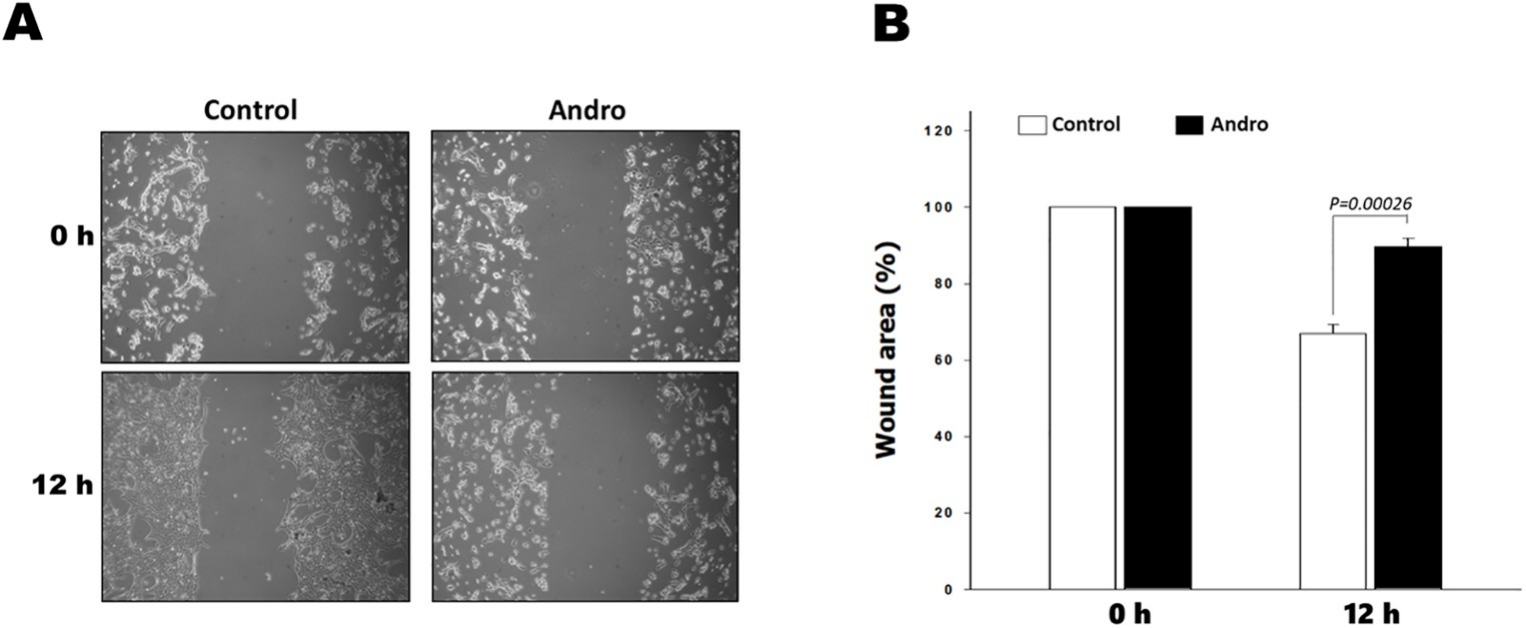
Andrographolide inhibits migration of RAS-transformed cells. Wound healing assay was evaluated after incubation with or without 10 uM andrographolide (Andro). Migration distance was measured at 0 and 12 hours after cells were scratched. (A) Wound healing assay under microscope. (B) The graphical data represented the percentage of migration area. Data are presented as the means± SD of three experiments. *P* values indicate the statistical significance between control and andrographolide -treated cells for 12 hours.

We firstly performed a wound healing assay to assess the effects of andrographolide on cell migration of Ras-transformed cells. The untreated cells displayed significant wound closure and tended to protrude into the wound site. However, the cells treated with andrographolide exhibited decreased migration ability when compared to the control cells (Fig 1A and 1B). These results imply that andrographolide inhibited the migration of Ras-transformed cells.

### Andrographolide with radiation inhibited Ras-transformed cell metastasis *in vivo*

To further determine whether andrographolide could reduce metastasis *in vivo*, xenograft tumor models were used in BALB/c nude mice. No significant alterations in body weight were detected following andrographolide and/or radiation treatment. Mice lung metastatic rates are presented in Table 1. The mice with tumors/mice injected ratio of vehicle, andrographolide (10 μM), radiation (2 Gy), and andrographolide (10 μM) plus radiation (2 Gy) was 75.0%, 58.3%, 50.0%, and 33.3%, respectively (Table 1). The results indicated that andrographolide combined with radiation demonstrated an inhibitive effect on cancer metastasis.

**Table 1:** Lung metastatic rate in mice.

Tumorigenesis in lung area		
Group	Mice with tumors/mice injected	Ratio (%)
D0	9/12	75.0
A0	7/12	58.3
D2	6/12	50.0
A2	4/12	33.3

A2, andrographolide (10 μM) plus radiation (2 Gy); A0, andrographolide (10 μM); D2, radiation (2 Gy); D0, DMSO (100 μl; control)

### Andrographolide combined with radiation was associated with reducing MMP-2 activation

**Fig 2:**
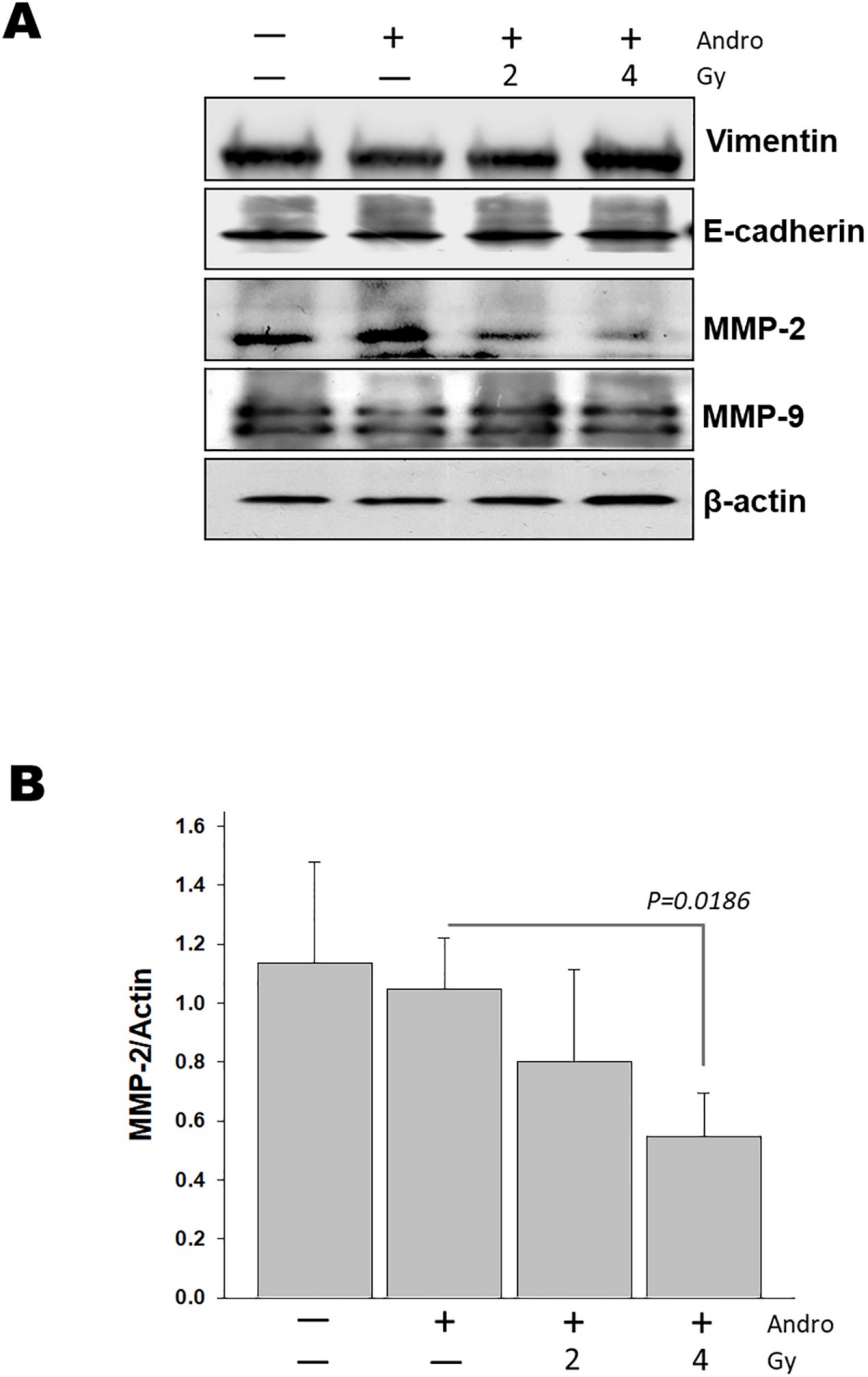
Effects of andrographolide and radiation on the expression EMT-related markers (E-cadherin, vimentin) and matrix metalloproteinases MMP-2 and -9. (A) RAS-transformed cells were treated with 10 uM andrographolide for 6 hours with and without 2 or 4 Gy radiation. After 24 hours, the cells were harvested for preparation of whole-cell protein lysates followed by Western blotting to detect the given proteins. (B) Quantitative analyses of MMP-2 expression evaluated by MMP-2/GAPDH ratio. The results are shown as mean ± SD (n = 3). *p < 0.05 for andrographolide combined with 4 Gy vs. andrographolide alone.

**Fig 3:**
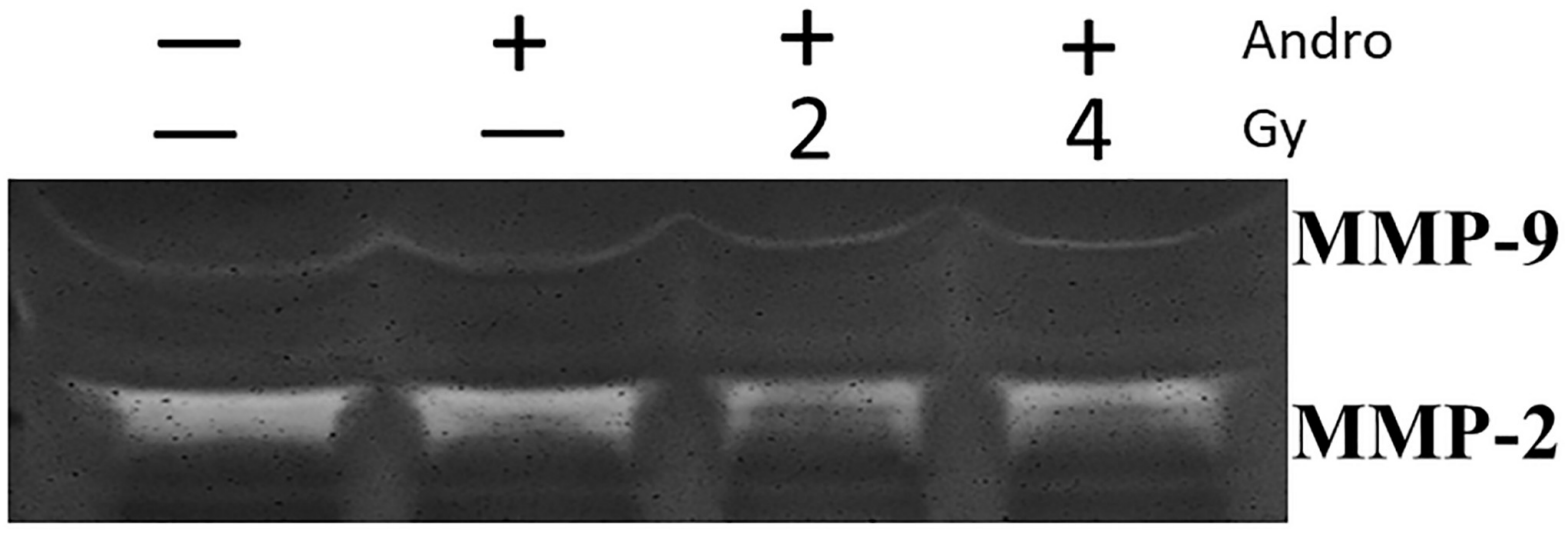
Andrographolide and radiation reduces MMP-2 expression. Gelatin zymography was performed using the conditioned media that were harvested after 48 hours in the presence or absence of 10 uM andrographolide, and then treated with/without radiation (2 or 4Gy) for 24 hours. The samples were applied without reduction to a 10% polyacrylamide gel containing gelatin, and proteolytic activity was demonstrated by digestion of the gelatin and clearing of the gel.

Epithelial-mesenchymal transition (EMT) is a developmental process, in which epithelial cells take on the characteristics of invasive mesenchymal cells and importantly determine the aggressiveness of cancer cells [27,28]. MMPs-dependent activation of the EMT program has been observed in a variety of cell types that involved in tumor development, cell proliferation, and metastatic dissemination [29,30]. Thus, we evaluated the effect of andrographolide with and without radiation on MMP-2, MMP-9, and EMT-related markers (i.e., E-cadherin, and vimentin protein) in Ras-transformed cells. Following treatment with andrographolide, the MMP-2 level was decreased significantly. Combination treatment of andrographolide and radiation more efficiently inhibited the expression of MMP-2 when compared with andrographolide alone. However, MMP-9, E-cadherin and vimentin protein levels were not altered by andrographolide alone or in combination with radiation (Fig 2A and 2B). To determine whether andrographolide regulates the expression of MMPs, we then measured MMP-2 and MMP-9 levels in the supernatant of *in vitro* cultured tumor cells by using gelatin zymography analysis. Andrographolide with radiation showed a significant reduction of MMP-2 enzyme activity, while MMP-9 activity was not significantly affected by the combined treatment (Fig 3). These data indicate that combined andrographolide and radiation synergistically inhibited MMP-2 activity.

### Andrographolide with radiation significantly inhibited MMP-2 activity through the suppression of ERK1/2 activation

**Fig 4:**
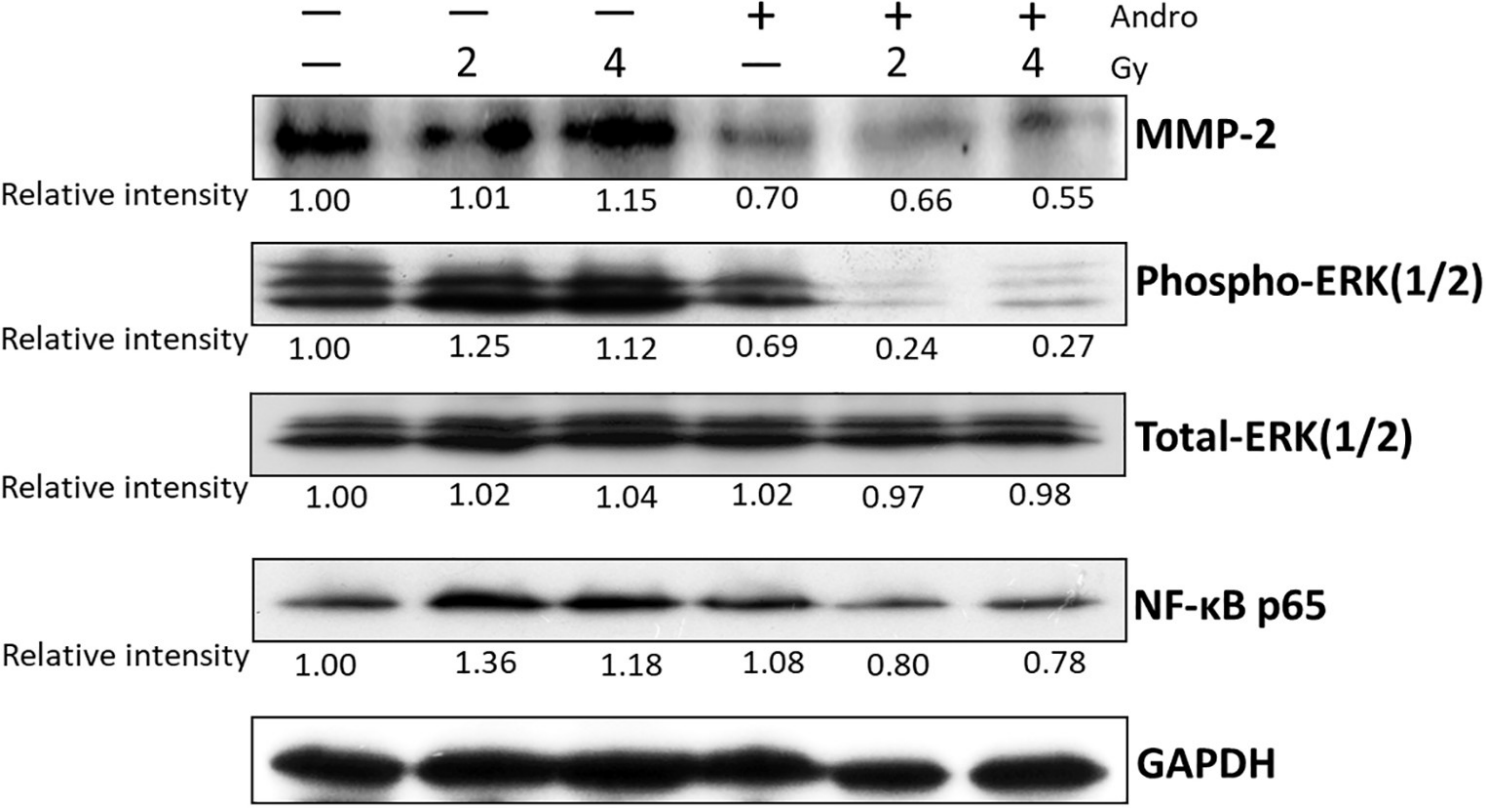
Andrographolide with radiation enhanced downregulation of radiation-induced MMP-2 leading to the suppression of ERK signaling. The cells were treated with 10 uM andrographolide with/without 2Gy or 4 Gy radiation for 24 hours. The figure displays typical data of MMP-2, phosphor-ERK1/2, total-ERK1/2 and NF-κB activity by Western blotting. Relative band intensities were analyzed by the Image J software. β-actin was used as an internal control for normalization. The numbers beneath the blots indicate the relative expression of each band when compared to the respective untreated control.

MMP-2 is a critical factor that regulates cancer metastasis, and appears to affect cell migration and invasion by affecting ERK1/2 and NF-κB signaling [31,32]. Our previous study found that radiation induced NF-κB activity in Ras-transformed cells, and andrographolide co-treatment reduced radiation-induced NF-κB activation [24]. Andrographolide has also been shown to have anti-invasive activity against colon cancer cells via inhibition of MMP-2 [20]. In addition, we examined whether the anti-metastatic effect of andrographolide combined with radiation is related to attenuation of the ERK1/2 or NF-κB signaling pathway. Expression of phospho-ERK 1/2 and NF-κB p65 were slightly increased in irradiated cells when compared with control cells, although MMP-2 was unchanged after radiation treatment. MMP-2 activity was inhibited in the presence of andrographolide, which was accompanied by reduction of phospho-ERK 1/2 and NF-κB p65 (Fig 4). Moreover, andrographolide combining with radiation more effectively suppressed MMP-2 and phospho-ERK 1/2 activity, but only slightly decreased activation of NF-κB p65 when compared with andrographolide treatment alone (Fig 4). These results demonstrate that the enhanced anti-metastatic effect of andrographolide in combination with radiation comes from inhibition of MMP-2 through modulation of ERK1/2 activity.

### Andrographolide with radiation inhibited cell invasion and this effect correlated with the down-regulation of MMP-2

**Fig 5:**
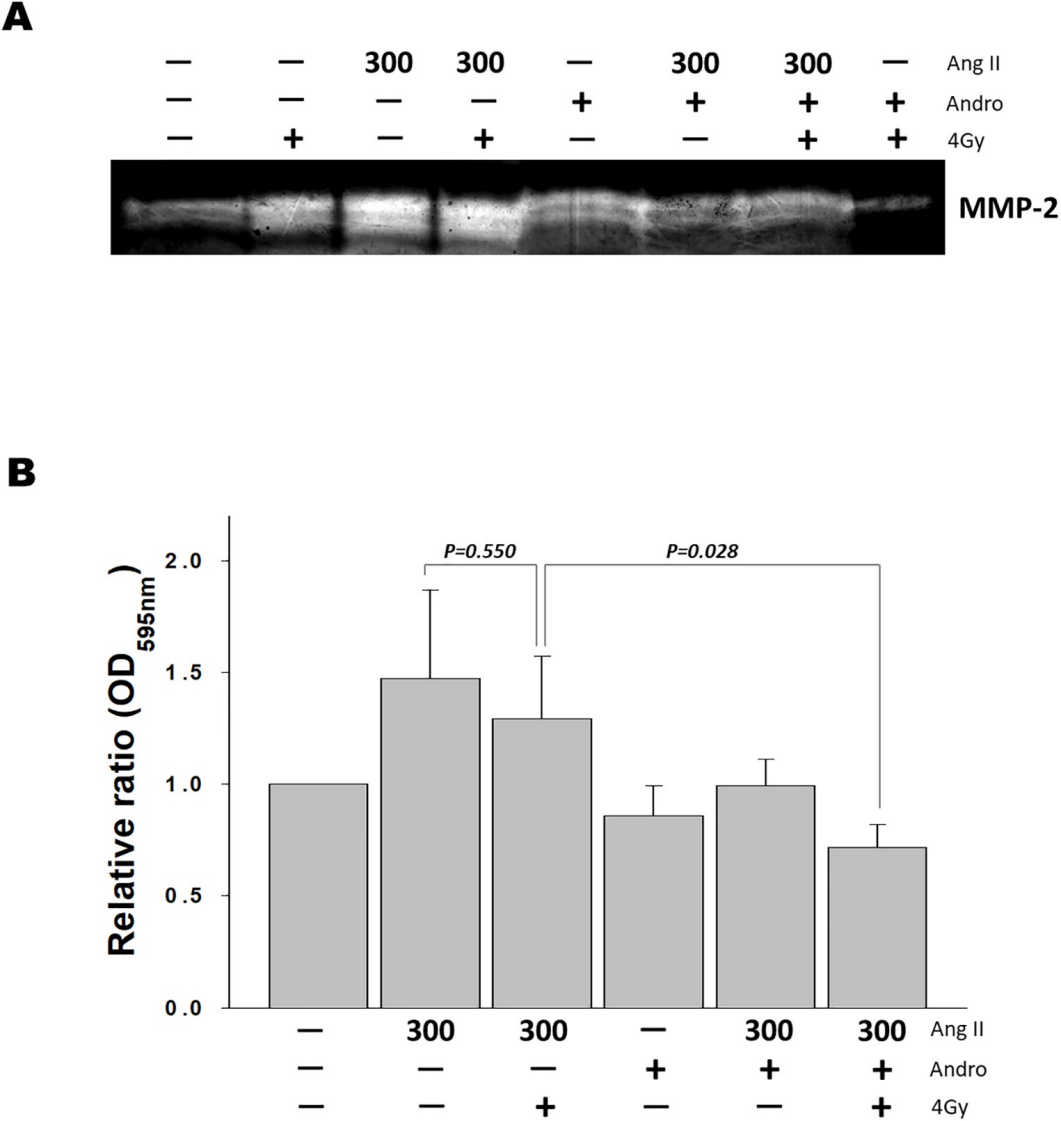
Andrographolide plus radiation reduced angiotensin II-induced MMP-2 activation and invasion. The Ras-transformed cells were incubated with/without 300 nM angiotensin II (Ang II) for 1 hour, and then treated with andrographolide, or radiation, or both for 24 hours. (A) The typical data show the gelatin zymography analysis band of the MMP-2 in conditioned media. (B) Invasion assays were performed using Transwell inserts of 8-micron pore size membrane and matrigel. After treatment, the cells were seeded in Transwell plates for 72 hours. The invaded cells were stained and quantified at an optical density of 560 nm. The experiments have been repeated three times; representative results of three independent experiments were shown. Quantification of cell invasion expressed as the percentage of control; one-way ANOVA was used for statistical analysis.

MMP-2 has been demonstrated to play an important role in mediating tumor invasion in many types of cancer cells [33]. Angiotensin II (Ang II) is a multifunctional octapeptide with diverse effects, including activation of MMPs while cell migration and invasion [34,35]. To further examine the effects of andrographolide on MMP-2 production, we investigated the effect of Ang II on MMP-2 expression in RAS-transformed cells. Being consistent with the Western blotting data, ionizing radiation increased the level of MMP-2 activity when compared with untreated cells, whereas the addition of andrographolide decreased MMP-2 expression. While MMP-2 activity was increased in the presence of Ang II, pretreatment with andrographolide caused an obvious decrease in Ang II-induced MMP-2 activity. Co-treatment with andrographolide and radiation more effectively enhanced the attenuating effects of Ang II-induced MMP-2 expression (Fig 5A). MMP-2 was reported to be closely associated with invasion and metastasis in several cancers. Consequently, we investigated the anti-invasive activities of Ras-transformed cancer cells upon exposure to either radiation or andrographolide in the presence of Ang II using Transwell assays. Compared with the control group, Ang II markedly facilitated invasion in Ras-transformed cells, whereas radiation treatment alone did not inhibit the Ang II-mediated response. In contrast, in the presence of andrographolide effectively inhibited Ang II-induced invasion. Notably, we observed that andrographolide combined with radiation more significantly suppressed cancer cell invasion when compared with treatment of radiation alone (Fig 5B). These results indicate that andrographolide effectively inhibits Ras-transformed cells invasion by suppressing MMP-2 activity, which might lead to the inhibition of metastasis.

### Andrographolide-mediated inhibition of MMP-2 activity via suppression of ERK signaling led to significant decrease cell migration and invasion of irradiated cells

**Fig 6:**
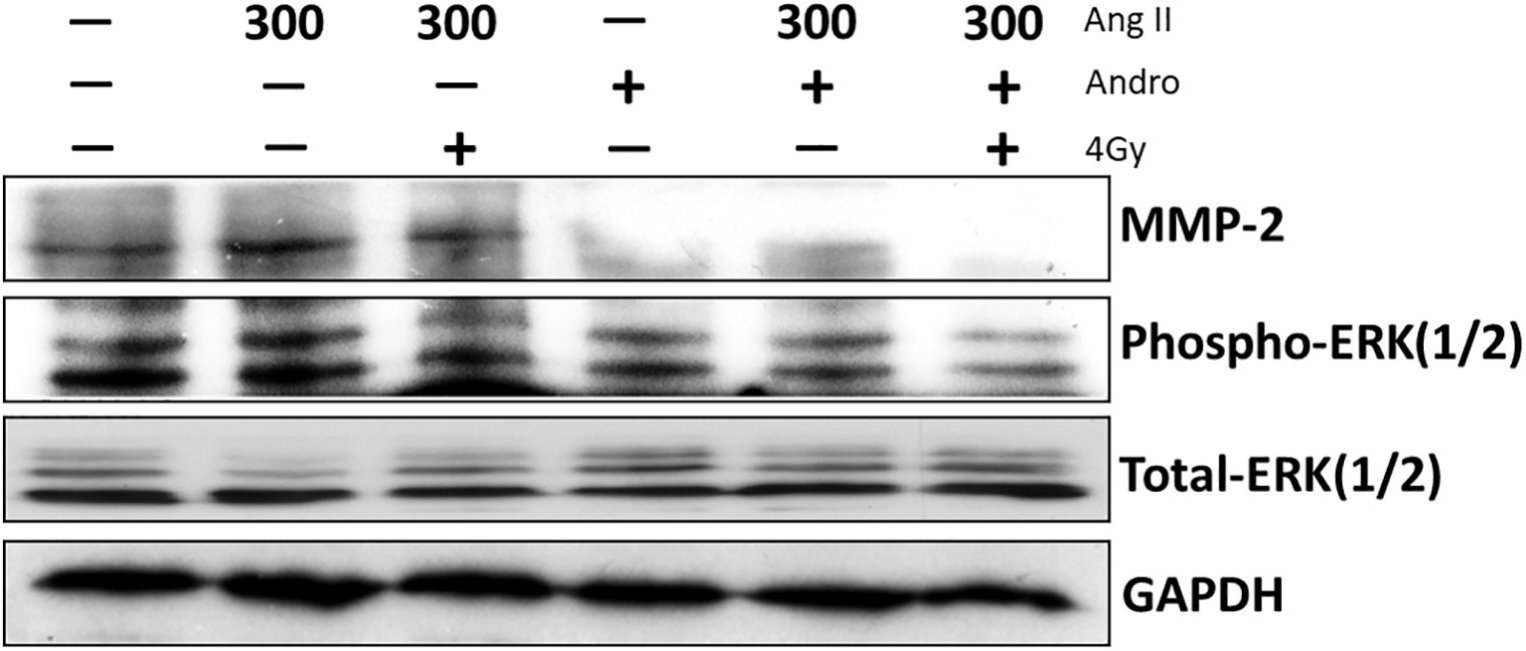
Andrographolide with radiation suppressed angiotensin II-induced MMP-2 expression through inhibition of ERK1/2 signaling. Cells were incubated with 300 nM Ang II for 1 hour and then treated with andrographolide, irradiation, or both for 24 hours. Protein level was determined by Western blotting.

Previous studies demonstrated that ERK1/2 is an important upstream factor in MMP-2 activation [35]. To confirm the mechanism andrographolide and MMP-2 regulation of cell migration and invasion, we next assessed the expression of phospho-ERK 1/2 of Ras-transformed cells after administration of andrographolide and/or radiation in the presence or absence of Ang II. As the above results, andrographolide could significantly attenuate MMP-2 up-regulation when compared with radiation alone. In addition, this effect was accompanied by significantly reducing phospho-ERK-1/2 activity. Andrographolide in combination with radiation resulted in a significant decrease in phospho-ERK-1/2 activity and MMP-2 level (Fig 6). Taken together, these results suggest that andrographolide combined with radiation greatly reduce MMP-2 expression by decreasing the activation of ERK1/2. Accordingly, andrographolide was able to down-regulate MMP-2 levels through inhibition of ERK1/2, leading to enhance the anti-growth and anti-metastatic effects of radiation in Ras-transformed cells.

## Discussion

RT is one of important treatments for cancer patients. Local recurrence and distance metastases, however, remain major obstacles to achieve a cure in patients with many solid tumors treated with RT. Therefore, investigating strategies to improve the sensitivity of cells to the cytotoxic effects of RT is required.

The *RAS* gene is known to be overstimulated in malignancies, and it is associated with an increased propensity for metastasis. Transfected Ras oncogenes have been shown to induce metastatic properties in some cells [36]. Additionally, overexpression of the Ras oncogene has been implicated in the development of radioresistance of various tumors [37,38], which can increase the risk of local recurrence after radiotherapy. Accordingly, in this study we used a Ras-transformed cell model to characterize the metastatic process and to explore the possible mechanism for the synergistic anti-metastatic effect of andrographolide in combination with RT.

Andrographolide and its derivatives have been shown to have anti-cancer activities in both *in vitro* and *in vivo* experimental models of cancer [39–41]. Recent studies demonstrated that andrographolide exerts inhibitory effects on malignant progression by repression of migration, matrix degradation, and invasion [42,43]. On the other hand, andrographolide also has been validated as a candidate for a radiosensitizing approach [24]. Studies have shown that andrographolide combined with RT have a synergistic anti-tumor effect in Ras-activated cancer cells [24,25]. We have further found that andrographolide enhanced the effectiveness of RT against Ras-dependent tumorigenesis. However, little is known about the mechanisms by which andrographolide enhances the sensitivity of cells to radiation. We found that andrographolide suppressed the migration and invasion of Ras-transformed cells. As a result, andrographolide may prevent cancer progression, such as metastasis. These results confirm our previous observations that andrographolide exerted marked inhibition of the metastasis of Ras-transformed cell *in vivo*.

MMP-2 and MMP-9 are major proteolytic enzymes contributed to ECM degradation to promote tumor cell migration and invasion [44–46]. Previous reports showed that radiation enhanced MMP-2 and MMP-9 secretion and activity, which enhanced tumor aggressiveness and promoted tumor metastasis [45,47]. We observed that ionizing radiation induced an increase in MMP-2 production but did not affect activation of MMP-9. MMP-2 is involved in tumor-induced and radiation-enhanced migration and invasion, and pre-treatment with andrographolide significantly decreased MMP-2 expression in irradiated cells, which reduced migration and invasion. On the other hand, EMT has been invoked as a critical component of the metastatic process, which endowed tumor cells with a more aggressive behavior [28,30,48]. In this study, we examined the levels of E-cadherin and vimentin (a mesenchymal marker) after treatment with either andrographolide alone or in combination with radiation. The results showed that E-cadherin and vimentin levels were not significantly different between the treatment and control groups.

Multiple signaling pathways, including ERK1/2 and NF-κB, have been associated with the pharmacological activity of andrographolide [13,49]. In addition, elevated basal NF-κB and ERK1/2 activity in certain cancers have been linked with tumor resistance to radiation, suggesting that radiation-induced activation of these kinase cascades may enhance the survival of irradiated cells [50,51]. Our prior study and other published reports have indicated that andrographolide significantly attenuates NF-κB activity, thus increasing radiation-induced cytotoxicity to tumor cells. In this study, we observed that radiation treatment alone induced NF-kB activity in Ras-transformed cells, and it might contribute to aggressive tumor growth. However, in the presence of andrographolide, radiation-induced NF-κB-dependent protein expression is inhibited.

ERK1/2 is a key molecule involved in the process of MAPKs pathway, which plays a crucial role in the progression of tumor cell proliferation and enhancs cancer survival after irradiation. Additionally, ERK1/2 has been shown to play a critical role in the regulation of cancer cell invasiveness by inducing MMPs-mediated extracellular matrix degradation [52,53]. Several lines of evidence also revealed that inhibition of ERK1/2 is associated with MMP-2 down-regulation to suppress cancer progression [20,54]. Furthermore, several studies showed that andrographolide reduces cancer invasion and metastatic abilities by inhibiting the activity of matrix metalloproteinases (MMPs) [20,42]. It showed that andrographolide suppresses the invasion ability of colon cancer cells via inhibition of MMP-2 activity and attenuation of the ERK signaling pathway [20]. Being consistent with these findings, our results indicated that andrographolide decreased MMP-2 activity, which was accompanied by a decrease of ERK1/2 phosphorylation, in Ras-transformed cells.

Further investigation showed that ERK1/2 is essential for MMPs expression mediated through activation of transcriptional factor NF-κB [55]. In this study, andrographolide treatment resulted in significant inhibition of MMP-2 expression, which was associated with slightly decreased NF-κB activation. More significantly, we observed that andrographolide inhibited radiation-induced MMP-2 expression, and was accompanied by the suppression of ERK1/2 activation. Combined treatment with andrographolide and radiation synergistically inhibited MMP-2 expression through the suppression of ERK1/2 signaling pathway in Ras-transformed cells could play an important role against the repression of metastasis. It has been demonstrated that Ang II induces expression of MMP-2 gene via ERK1/2 signaling pathway in different cell types [35]. In order to confirm our results, we further examined the effect of andrographolide on Ang II-induced MMP-2 in Ras-transformed cells. Andrographolide significantly attenuated Ang II-induced MMP-2 and related to diminished ERK1/2 activity in irradiated cells. Targeting and inhibiting both tumorigenesis and metastasis are the main strategies to increase the effectiveness of cancer treatments. Although targeted drugs are still developed in conjunction with RT to improve therapeutic efficacy in cancer patients, these agents are less effective for the prevention of metastasis. We have previously shown that andrographolide was able to enhance tumor regression in conjunction with ionizing radiation in the Ras-transformed model *in vitro* and *in vivo* [24]. Moreover, andrographolide combined with RT has been shown to have a synergistic effect that significantly inhibits cancer metastasis *in vivo*. Our findings indicate that andrographolide with radiation attenuates oncogenic RAS-expressing cell migration and invasion, which is mediated downregulation of activated MMP2 with the suppression of ERK1/2 signaling. The results suggest that andrographolide may be an effective strategy for overcoming radiation-induced cancer progression and metastasis.
